# Supplementary material for: Bridging the Chemical Profiles and Biological Effects of Spathodea campanulata Extracts: A New Contribution on the Road from Natural Treasure to Pharmacy Shelves
Source: Molecules. 2022 Jul 22;27(15):4694. doi: 10.3390/molecules27154694 (PMC9330408; doi:10.3390/molecules27154694)
Supplement: Supplementary file 1 [file molecules-27-04694-s001.zip › molecules-1829459-supplementary.pdf]

Article

Bridging the chemical profiles and biological effects of *Spathodea campanulata* extracts: A new contribution on the road from natural treasure to pharmacy shelves

Łukasz Świątek[1*], Elwira Sieniawska[2], Kouadio Ibrahime Sinan[3], Gokhan Zengin[3*], Abdullahi Ibrahim Uba[4], Kouadio Bene[5], Magdalena Maciejewska-Turska[6], Barbara Rajtar[1], Małgorzata Polz-Dacewicz[1], Abdurrahman Aktumsek[3]

[1]Department of Virology with SARS Laboratory, Medical University of Lublin, Chodzki 1, 20-093 Lublin, Poland. lukasz.swiatek@umlub.pl (L.S); barbara.rajtar@umlub.pl (B.R); malgorzata.polz-dacewicz@umlub.pl (M.P)

[2]Department of Natural Products Chemistry, Medical University of Lublin, 20-093 Lublin, Poland. esieniawska@pharmacognosy.org (E.S),

[3]Department of Biology, Science Faculty, Selcuk University, 42130 Konya, Turkey. sinankouadio@gmail.com (K.I.S); gokhanzengin@selcuk.edu.tr (G.Z); aktumsek@selcuk.edu.tr (A.A)

[4] Department of Molecular Biology and Genetics, Faculty of Engineering and Natural Sciences, Kadir Has University, Istanbul 34083, Turkey. abdullahi.iu2@gmail.com (A.I.B)

[5]Laboratoire de Botanique et Phytothérapie, Unité de Formation et de Recherche Sciences de la Nature, Université Nangui Abrogoua, 02 BP 801 Abidjan 02, Ivory Coast. kouadio777@gmail.com (K.B)

[6]Department of Pharmacognosy with Medicinal Plants Garden, Medical University of Lublin, 20-093 Lublin, Poland magdalena.maciejewska@umlub.pl (Ö.M)

* Correspondence: lukasz.swiatek@umlub.pl and gokhanzengin@selcuk.edu.tr

Cell line maintenance and in vitro experiments

The cytotoxicity of *Spathodea campanulata* extracts was evaluated *in vitro* towards normal VERO (ECACC, No. 84113001) cells and cancer-derived cell lines – FaDu (ATCC, HTB-43, human hypopharyngeal squamous cell carcinoma), HeLa (ECACC, Cat. No. 93021013, cervical adenocarcinoma) and RKO (ATCC, Cat. No. CRL-2577, colon carcinoma), using 3-(4,5-dimethylthiazol-2-yl)-2,5-diphenyltetrazolium bromide (MTT) based protocol.

Media used for *in vitro* culturing included Dulbecco Modified Eagle Medium (DMEM, Corning, Tewksbury, MA, USA) used for VERO cells and Modified Eagle Medium (MEM, Corning) used for other cell lines. Cell media used in the experiments were supplemented with antibiotics (Penicillin-Streptomycin Solution, Corning) and fetal bovine serum (FBS, Corning) – 10% (cell passaging) and 2% (cell maintenance and experiments). Phosphate buffered saline (PBS) and trypsin were bought from Corning, whereas MTT (3-(4,5-dimethylthiazol-2-yl)-2,5-diphenyltetrazolium bromide) and DMSO (dimethyl sulfoxide) from Sigma (Sigma-Aldrich, St. Louis, MO, USA). Incubation was carried out in a 5% CO_2 atmosphere at 37°C (CO_2 incubator, Panasonic Healthcare Co., Tokyo, Japan).

The cytotoxicity testing

Cytotoxicity was tested using an MTT-based protocol following a previously described protocol [85]. Briefly, the cells were passaged into 96-well plates (Falcon, TC-treated, Corning) and, after overnight incubation, treated with serial dilutions of extract stock solutions for 72 h. Afterwards, the media was removed, cells were washed with PBS, and 10% of MTT solution (5 mg/mL) in cell media was added, and the incubation continued for the next 4 h. Subsequently, the SDS/DMF/PBS (14% SDS, 36% DMF, 50%

PBS) solvent was used (100 µL per well) to dissolve the precipitated formazan crystals and the plates were left at 37°C overnight. Finally, the Synergy H1 Multi-Mode Microplate Reader (BioTek Instruments, Inc. Winooski, Vermont, USA) with Gen5 software (ver. 3.09.07; BioTek Instruments, Inc.) was used to measure the absorbance (540 and 620 nm).

Evaluation of the antiviral properties

The antiviral activity of *Spathodea campanulata* extracts was tested against HHV-1 (ATCC, Cat. No. VR-260) and CVB3 (ATCC No. VR-30, Coxsackievirus B3) propagated in the VERO cell line. The antiviral assays involved the influence of extracts on the formation of virus (HHV-1 or CVB3) induced cytopathic effect (CPE) and the evaluation of the reduction of infectious titer using the end-point virus titration and the viral load using Real-Time PCR.

Evaluation of the influence on the virus-induced CPE

The infectious titer of HHV-1 used in this study was 5.5±0.25 log$CCID_{50}$/mL ($CCID_{50}$ – 50% cell culture infectious dose). Briefly, the VERO cells (monolayer) in 48-well plates (Falcon, clear flat bottom TC-treated, Corning) were treated (500 µL/well) with HSV-1 or CVB3 suspension (100* $CCID_{50}$/mL) in cell media and incubated for 1 hour, leaving at least two uninfected wells as VERO cell control. Afterwards, the media were removed, monolayers washed with PBS, and the non-toxic concentrations of extracts, the highest concentration not exceeding the CC_{10} values, diluted in cell media were added. The non-infected VERO cells (cell control) and non-treated infected cells (virus control) wells were maintained in media containing 2% FBS. The incubation was conducted until cytopathic effect (CPE) was observed (inverted microscope CKX41, Olympus Corporation,

Tokyo, Japan) in virus control, usually approx. 72h. Afterwards, the plates were observed for possible inhibition of CPE by tested extracts compared to the CPE in virus control, and the results were recorded. Lastly, the plates were thrice frozen (-72°C) and thawed; the samples were collected and stored at -72°C until used in the end-point virus titration assay and DNA isolation. Antiviral properties of extracts were tested in three independent experiments.

End-point dilution assay for HHV-1 titration

Samples collected from antiviral assays were subjected to an end-point dilution assay to evaluate the HHV-1 or CVB3 titers. Briefly, the VERO cells (monolayer) in 96-well plates were incubated with ten-fold dilutions of samples (3 replicates) in cell media for 72 hours. The daily observation was conducted to monitor the development of CPE. After the incubation, all media were removed and the virus infectious titer for each sample was measured using the previously described MTT method. Subsequently, the difference (Δlog) of HHV-1 infectious titer ($logCCID_{50}$/mL) in the samples treated with tested *Spathodea campanulata* extracts (FE) and in the virus control (VC) from the same experiment ($\Delta log = logCCID_{50}VC - logCCID_{50}FE$) were calculated. The Δlog values were evaluated for every antiviral assay, and the results were expressed further as means of viral titer reduction. A significant antiviral activity can be reported for extracts decreasing the infectious titer by at least 3 log compared to virus control [85].

Real-Time PCR for HHV-1 viral load

The DNA isolation was carried out using a commercially available kit (QIAamp DNA Mini Kit, Cat#51304, QIAGEN GmbH, Hilden, Germany) following the manufacturer's instructions. The Real-Time PCR amplification was performed using HOT FIREPol

EvaGreen qPCR Mix (Solis BioDyne, Tartu, Estonia) and primers (UL54F – 5' CGCCAAGAAAATTTCATCGAG 3', UL54R – 5' ACATCTTGCACCACGCCAG 3') on the CFX96 thermal cycler (Bio-Rad Laboratories, Inc., California, USA). The amplification cycle parameters were as follows: initial activation (95°C, 15 min); cycling (40 repeats: denaturation (95°C, 15 secs), annealing (60°C, 20 secs), and synthesis (72°C, 20 secs), fluorescence acquisition); melting curve analysis (60-95°C). The quantitative analysis was carried out using a calibration curve comprised of tenfold dilutions of HHV-1 DNA isolate, which were previously quantitatively analysed using IVD certified GeneProof Herpes Simplex Virus (HSV-1/2) PCR Kit (Cat#HSV/ISEX/025, GeneProof a.s., Czech Republic).

Supplementary material references

[85] Świątek, Ł.; Sieniawska, E.; Sinan, K.I.; Maciejewska-Turska, M.; Boguszewska, A.; Polz-Dacewicz, M.; Senkardes, I.; Guler, G.O.; Bibi Sadeer, N.; Mahomoodally, M.F.; Zengin, G. LC-ESI-QTOF-MS/MS Analysis, Cytotoxic, Antiviral, Antioxidant, and Enzyme Inhibitory Properties of Four Extracts of *Geranium pyrenaicum* Burm. f.: A Good Gift from the Natural Treasure. *Int. J. Mol. Sci.* 2021, *22*, 7621. https://doi.org/10.3390/ijms22147621

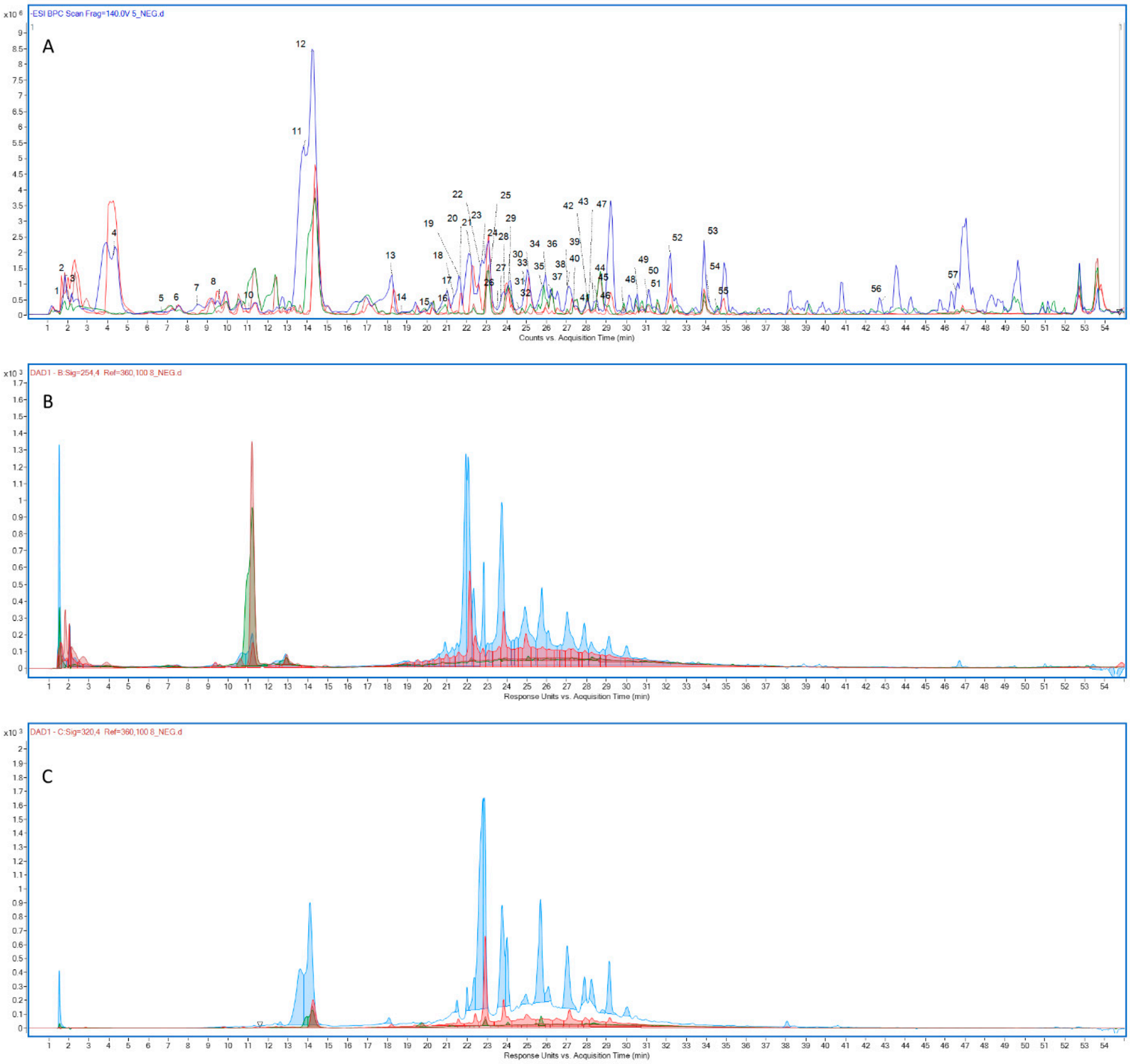

Figure S1. Spectrochromatographic profiles of *S. campanulata* extracts: A – Base peak chromatograms, B – UV chromatograms 254 nm, C – UV chromatograms 320 nm, blue – leaves-MeOH, red – leaves-infusion, green – stem bark-MeOH, brown – stem bark-infusion, samples analyzed at the concentration of 20 mg/mL
